# Supplementary material for: Mechanisms governing the pioneering and redistribution capabilities of the non-classical pioneer PU.1
Source: Nat Commun. 2020 Jan 21;11:402. doi: 10.1038/s41467-019-13960-2 (PMC6972792; doi:10.1038/s41467-019-13960-2)
Supplement: Supplementary file 7 — Source data [file 41467_2019_13960_MOESM7_ESM.zip › Source_Data/Figure6/Figure6B_MotifScanOutput/FLI1_mutPU.1/homerResults/motif35.similar.html]

motif35

## Information for motif35

T
C
A
G
G
A
T
C
G
T
A
C
C
T
G
A
T
A
G
C
A
G
T
C
C
G
T
A
A
C
T
G
C
T
G
A
C
A
T
G
T
C
A
G
C
T
A
G
G
T
A
C
C
T
A
G
  
Reverse Opposite:  

G
A
T
C
C
A
T
G
A
G
T
C
A
G
T
C
G
T
A
C
G
A
C
T
A
G
T
C
G
A
C
T
T
C
A
G
A
T
C
G
G
A
C
T
A
C
T
G
C
T
A
G
A
G
T
C
  

|  |  |
| --- | --- |
| p-value: | 1e-31 |
| log p-value: | -7.162e+01 |
| Information Content per bp: | 1.677 |
| Number of Target Sequences with motif | 179.0 |
| Percentage of Target Sequences with motif | 1.74% |
| Number of Background Sequences with motif | 188.8 |
| Percentage of Background Sequences with motif | 0.50% |
| Average Position of motif in Targets | 98.3 +/- 59.6bp |
| Average Position of motif in Background | 97.7 +/- 62.0bp |
| Strand Bias (log2 ratio + to - strand density) | -0.2 |
| Multiplicity (# of sites on avg that occur together) | 1.02 |
| Motif File: | file (matrix) reverse opposite |

### Similar de novo motifs found

|  |  |  |  |  |  |  |  |
| --- | --- | --- | --- | --- | --- | --- | --- |
| Rank | Match Score | Redundant Motif | P-value | log P-value | % of Targets | % of Background | Motif file |
| 1 | 0.926 | G T A C G T A C T C G A T A G C G C A T C T G A A C T G C T A G A C T G C T A G A T C G A G T C C T A G | 1e-29 | -68.383491 | 2.15% | 0.74% | motif file (matrix) |
| 2 | 0.906 | G A T C A C T G A G T C A T G C A G T C A G C T A G T C A G C T C T G A A T C G A G C T A T C G | 1e-24 | -55.853555 | 1.86% | 0.67% | motif file (matrix) |
| 3 | 0.812 | C A T G A G T C T A G C G T A C G A T C A G T C A G C T C T G A A T C G A G C T A C T G | 1e-23 | -53.400563 | 1.83% | 0.67% | motif file (matrix) |
| 4 | 0.784 | G A T C C A T G T G A C A G T C A T G C G C A T A T G C C G A T T C G A T A C G | 1e-21 | -50.478305 | 1.44% | 0.47% | motif file (matrix) |
